# p45 NF-E2 regulates syncytiotrophoblast differentiation by post-translational GCM1 modifications in human intrauterine growth restriction

**DOI:** 10.1038/cddis.2017.127

**Published:** 2017-04-06

**Authors:** Shrey Kohli, Juliane Hoffmann, Franziska Lochmann, Paulina Markmeyer, Hanna Huebner, Fabian B Fahlbusch, Moh'd Mohanad Al-Dabet, Ihsan Gadi, Jayakumar Manoharan, Michael Löttge, Ana C Zenclussen, Anat Aharon, Benjamin Brenner, Khurrum Shahzad, Matthias Ruebner, Berend Isermann

**Affiliations:** 1 Institute of Clinical Chemistry and Pathobiochemistry, Otto-von-Guericke-University Magdeburg, Magdeburg, Germany; 2 Health Campus Immunology, Infectiology and Inflammation, Otto-von-Guericke-University, Magdeburg, Germany; 3 Department of Gynaecology and Obstetrics, Comprehensive Cancer Center Erlangen-FMN, Friedrich-Alexander University Erlangen-Nuremberg, Erlangen, Germany; 4 Department of Pediatrics and Adolescent Medicine, Friedrich-Alexander University Erlangen-Nuremberg, Erlangen, Germany; 5 Gynecology and Obstetrics, Klinikum Magdeburg, Magdeburg, Germany; 6 Experimental Obstetrics and Gynecology, Medical Faculty, Otto-von-Guericke University, Magdeburg, Germany; 7 Department of Hematology, Rambam Health Care Campus, Haifa, Israel; 8 Department of Biotechnology, University of Sargodha, Pakistan

## Abstract

Placental insufficiency jeopardizes prenatal development, potentially leading to intrauterine growth restriction (IUGR) and stillbirth. Surviving fetuses are at an increased risk for chronic diseases later in life. IUGR is closely linked with altered trophoblast and placental differentiation. However, due to a paucity of mechanistic insights, suitable biomarkers and specific therapies for IUGR are lacking. The transcription factor p45 NF-E2 (nuclear factor erythroid derived 2) has been recently found to regulate trophoblast differentiation in mice. The absence of p45 NF-E2 in trophoblast cells causes IUGR and placental insufficiency in mice, but mechanistic insights are incomplete and the relevance of p45 NF-E2 for human syncytiotrophoblast differentiation remains unknown. Here we show that p45 NF-E2 negatively regulates human syncytiotrophoblast differentiation and is associated with IUGR in humans. Expression of p45 NF-E2 is reduced in human placentae complicated with IUGR compared with healthy controls. Reduced p45 NF-E2 expression is associated with increased syncytiotrophoblast differentiation, enhanced glial cells missing-1 (GCM1) acetylation and GCM1 desumoylation in IUGR placentae. Induction of syncytiotrophoblast differentiation in BeWo and primary villous trophoblast cells with 8-bromo-adenosine 3′,5′-cyclic monophosphate (8-Br-cAMP) reduces p45 NF-E2 expression. Of note, p45 NF-E2 knockdown is sufficient to increase syncytiotrophoblast differentiation and GCM1 expression. Loss of p45 NF-E2 using either approach resulted in CBP-mediated GCM1 acetylation and SENP-mediated GCM1 desumoylation, demonstrating that p45 NF-E2 regulates post-translational modifications of GCM1. Functionally, reduced p45 NF-E2 expression is associated with increased cell death and caspase-3 activation *in vitro* and in placental tissues samples. Overexpression of p45 NF-E2 is sufficient to repress GCM1 expression, acetylation and desumoylation, even in 8-Br-cAMP exposed BeWo cells. These results suggest that p45 NF-E2 negatively regulates differentiation and apoptosis activation of human syncytiotrophoblast by modulating GCM1 acetylation and sumoylation. These studies identify a new pathomechanism related to IUGR in humans and thus provide new impetus for future studies aiming to identify new biomarkers and/or therapies of IUGR.

## Key Points:

- Expression of the transcription factor p45 NF-E2 is reduced in human IUGR placentae compared with healthy controls.
- Loss of p45 NF-E2 is sufficient to induce syncytiotrophoblast differentiation.
- p45 NF-E2 deficiency promotes CBP-mediated GCM1 acetylation and SENP-mediated GCM1 desumoylation, enhancing GCM1 transcriptional activity and syncytiotrophoblast differentiation.
- Reduced p45 NF-E2 expression in human IUGR is associated with increased GCM1 acetylation and GCM1 desumolyation, corroborating the translational relevance of the current findings.

Placental insufficiency is a frequent cause of perinatal morbidity and mortality, occurring in about 5–7% of pregnancies.^1, 2^ Placental insufficiency can manifest as a spectrum of disorders, including intrauterine growth restriction (IUGR), abruption and stillbirth. Besides, placental insufficiency predisposes the newborn to diseases in later life such as diabetes mellitus or cardiovascular complications.^3, 4, 5, 6^ Suitable biomarkers and efficient therapies are currently lacking. Accordingly medical management of IUGR remains a challenge.^1^ While preterm delivery of the fetus may prevent further deterioration of the IUGR and associated risks, it carries itself substantial health risks for the fetus. Additionally, developmental impairment, for example of the brain, may already be present at this time. Hence, biomarkers for early detection of placental insufficiency aiding physicians in decision making are needed. Furthermore, the need for potential therapeutic approaches requires new mechanistic insights into causes of IUGR.

Impaired trophoblast differentiation is closely associated with disturbed placentation and pregnancy-associated diseases such as IUGR.^7^ Glial cells missing-1 (GCM1) is an important regulator of trophoblast differentiation, turnover and maintenance. Both reduced and increased levels of GCM1 have been described in human pregnancy complications^8, 9^ and have been linked with altered trophoblast function *in vitro.*^10^ GCM1 is best known for its function as a regulator of syncytiotrophoblast differentiation, controlling the expression of the fusogenic genes syncytin-1 and syncytin-2 among others.^11, 12^ GCM1 is tightly regulated by post-translational modifications. Thus, acetylation of GCM1 increases its stability and transcriptional activity, while sumoylation inhibits the transcriptional activity of GCM1.^13^ However, studies evaluating the regulation of GCM1 were mostly limited to *in vitro* work and the functional relevance and (patho-) physiological regulators of GCM1 in human placental disease remain incompletely defined, hampering translational efforts.

Using approaches in mice and *in vitro* with mouse-derived trophoblast cells we previously identified a new function of the transcription factor p45 NF-E2 (nuclear factor erythroid derived 2) in placental development and function. The transcription factor NF-E2 belongs to the basic leucine-zipper family of transcription factors and is composed of a heterodimer formed of a tissue-restricted 45 kDa (p45 NF-E2) and widely expressed 18 kDa (p18, including MafF, MafG and MafK) subunits.^14^ The *in vivo* role of NF-E2 was studied in mice lacking the p45 subunit, which was thought to be restricted to hematopoietic cells and to be of uttermost importance for erythropoiesis. Unexpectedly, the mice displayed only a mild defect in erythropoiesis but a severe impairment of megakaryopoiesis, resulting in severe thrombocytopenia with a near complete absence of normal platelets, and an associated IUGR.^15^ The IUGR in p45 NF-E2-deficient embryos is independent of thrombocytopenia and detailed mechanistic studies revealed a new function of p45 NF-E2 in trophoblast cell differentiation.^16, 17^ In the absence of p45 NF-E2 enhanced GCM1 activity and syncytiotrophoblast formation impairs placental vascularization and embryonic growth in mice. While these studies established a novel function p45 NF-E2 for syncytiotrophoblast differentiation through regulation of GCM1, the mechanism through which p45 NF-E2 regulates GCM1 remained unknown. Likewise, it remains unknown whether other post-translational modifications of GCM1, which modulate GCM1 activity, are regulated by p45 NF-E2 and – importantly – the relevance of these findings for human trophoblast cells and placental disease in humans remains unknown.

To address these open questions we analyzed placental tissues from human uncomplicated (control) pregnancies or pregnancies complicated by IUGR and we employed an *in vitro* human trophoblast cell line model to study the relevance and mechanisms of p45 NF-E2-dependent trophoblast differentiation. We establish a functional role of p45 NF-E2 for syncytiotrophoblast differentiation in human trophoblast cells. Syncytiotrophoblast formation in human trophoblast cells is regulated by p45 NF-E2 via CREB-binding protein (CBP)-dependent GCM1 acetylation and SENP1-mediated GCM1 desumoylation. The relevance of these findings is corroborated by corresponding observations made in human tissues samples. These results are expected to foster efforts to establish biomarkers and new therapeutic strategies for IUGR.

## Results

### Reduced placental expression of p45 NF-E2 in human IUGR

**Figure 1:**
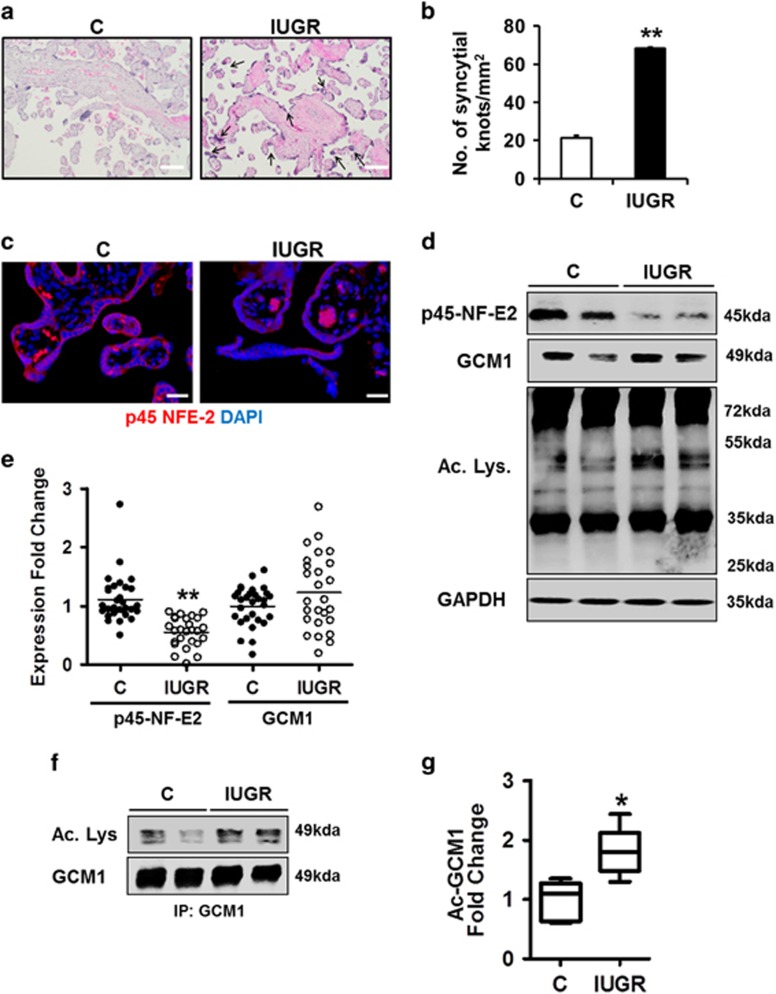
Altered p45 NF-E2 expression and GCM1 acetylation in human IUGR placentae**.** (**a** and **b**) Increased syncytial knot formation/mm^2^ in IUGR placentae compared with controls (H&E staining; (a) representative images; (b) bar graph summarizing results). (**c**) p45 NF-E2 in downregulated in placentae obtained from women with pregnancies complicated with IUGR compared with controls (representative immunofluorescence images; red: p45 NF-E2; blue: DAPI nuclear staining). (**d** and **e**) Reduced expression of p45 NF-E2 and enhanced lysine-acetylation in placenta tissue lysates obtained from women with pregnancies complicated with IUGR compared with controls. GCM1 expression did not differ significantly between the two cohorts (immunoblot analysis; (**d**), representative immunoblots; (**e**), dot-plot summarizing results). (**f** and **g**) Gcm1 acetylation is increased in placenta tissue lysates obtained from women with pregnancies complicated with IUGR compared with controls; immunoprecipitation using anti-GCM1 antibody followed by immunoblotting ((**f**) representative immunoblots; (**g**) box-plot summarizing results). Size bars represent 100 *μ*m (**a**) and 20 *μ*m (**c**); mean±S.E.M. (**b**,**e** and **g**); *P<0.05, **P<0.01 (*t*-test in **b** and **g**; Mann–whitney *U*-test in **e**)

The absence of p45 NF-E2 in mouse placenta impairs placental vascularization and results in IUGR in mice.^18^ In order to ascertain the relevance of p45 NF-E2 for placental function and syncytiotrophoblast differentiation in humans, we analyzed human placentae from 26 women with normotensive IUGR pregnancies and no indication of preeclampsia along with control placentae from 30 women with normal pregnancies. Histological assessment by hematoxylin and eosin (H&E) staining revealed excess syncytial knot formation in placentae obtained from pregnancies complicated by IUGR (Figures 1a and b). Immunofluorescence and immunoblotting analysis of these placentae revealed reduced p45 NF-E2 expression in placentae from pregnancies complicated by IUGR compared with controls (Figures 1c–e). Thus, IUGR and excess syncytium formation, as reflected by an increase of syncytial knots, are associated with reduced p45 NF-E2 expression in humans, suggesting that the observations made in mice and the mechanistic deduction may be also relevant for human pregnancies associated with IUGR.

### GCM1 acetylation is induced in human IUGR

While in the murine model of IUGR reduced p45 NF-E2 expression was linked with increased GCM1 expression, the expression of GCM1 did not differ between human placentae obtained from pregnancies complicated by IUGR and healthy control placentae (Figures 1d and e). Of note, not only expression but also acetylation of GCM1 was altered in p45 NF-E2−/− murine placentae.^18^ Post-translational modifications of GCM1 such as acetylation are known to control the GCM1 stability and activity, and thus trophoblast differentiation via trophoblast cell fusion.^19, 20^ This raises the question as to whether p45 NF-E2 regulates GCM1 activity in human placentae primarily via post-translational modifications such as acetylation. Given our and others previous results^18, 19, 20^ we next analyzed protein acetylation in general and specifically that of Gcm1 in human placental tissues. Analyses of protein acetylation using an antibody against acetylated lysine revealed increased acetylation of some proteins, including proteins matching the size of GCM1 (49 kDa; Figure 1d). Increased acetylation of GCM1 in placentae from pregnancies complicated by IUGR was confirmed by immunoprecipitation (Figures 1f and g).

Taken together, these findings demonstrate that increased GCM1 acetylation is readily detectable in human placentae from pregnancies complicated by IUGR, suggesting that p45 NF-E2 regulates GCM1 acetylation in human trophoblast cells.

### p45 NF-E2 regulates syncytiotrophoblast differentiation in human trophoblast cells

**Figure 2:**
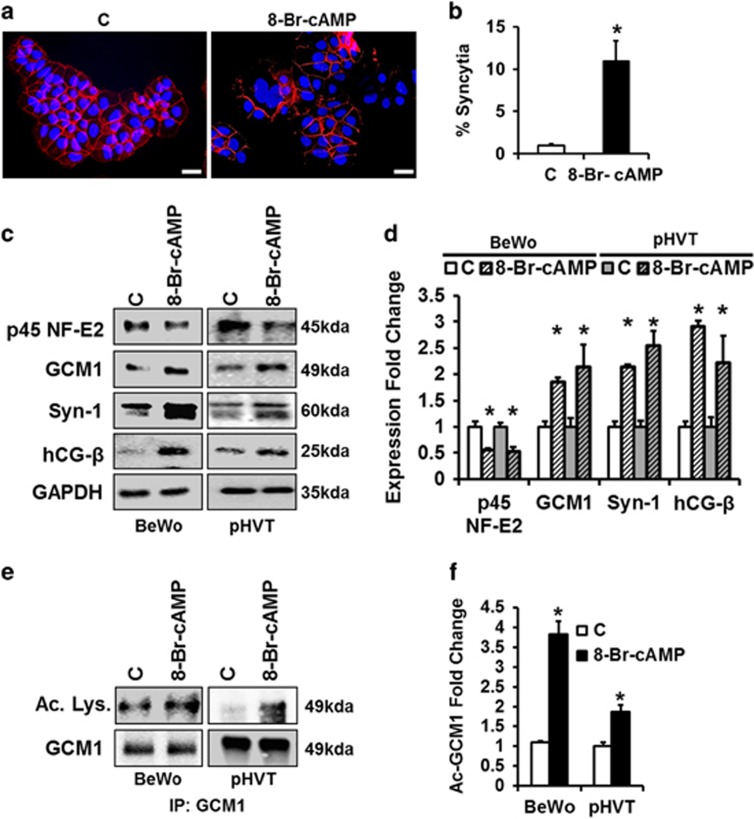
p45 NF-E2 regulates syncytiotrophoblast differentiation of human trophoblast cells *in vitro.*(**a** and **b**) Enhanced syncytia formation after 8-Br-cAMP treatment in BeWo cells; immunofluorescence staining ((**a**) representative images, red: E-cadherin, blue: DAPI nuclear staining; (**b**) bar graph summarizing results). (**c** and **d**) Downregulation of p45 NF-E2 expression after 8-Br-cAMP treatment in BeWo cells and primary human villous trophoblast cells (pHVT) accompanied by upregulation of differentiation markers GCM1, Syn-1 and hCG-β expression, indicating increased syncytiotrophoblast formation compared with control BeWo cells (immunoblotting analysis; (**c**) representative immunoblots; (**d**) bar graph summarizing results). (**e** and **f**) 8-Br-cAMP induces acetylation of GCM1. Immunoprecipitation using anti-GCM1 antibody followed by immunoblotting for acetylated lysine ((**e**) representative immunoblots; (**f**) bar graph summarizing results) showing enhanced GCM1 acetylation after treatment of BeWo cells and primary human villous trophoblast cells (pHVT) with 8-Br-cAMP. Size bars represent 50 *μ*m (**a**); mean±S.E.M. (**b**,**d** and **f**); results of at least five independent repeat experiments shown (**b**,**d** and **f**); *P<0.05 (*t*-test in **b**,**d** and **f**)

To ascertain the mechanism through which p45 NF-E2 controls syncytium formation in human trophoblast cells, including the potential role of GCM1 regulation via post-translational modifications, we first established a suitable *in vitro* model using the trophoblast-derived cell line BeWo. BeWo cells were differentiated using 8-Br-cAMP to induce syncytiotrophoblast formation.^21^ Treatment of BeWo cells with 8-Br-cAMP resulted in formation of multinucleate syncytia, as determined by immunostaining for E-cadherin and DAPI nuclear counterstain (Figures 2a and b). Trophoblast syncytium formation was confirmed by increased expression of the syncytiotrophoblast differentiation markers human chorionic gonadotropin β (hCG-β) and Syncytin-1 (Syn-1) (Figures 2c and d). Intriguingly, in this *in vitro* model GCM1 expression was increased, consistent with increased syncytiotrophoblast differentiation and the observations made in murine trophoblast cells.^18^ BeWo cells, although frequently used to study trophoblast differentiation and syncytialization, are choriocarcinoma-derived cells and accordingly have limitations. To validate our approach we used primary villous trophoblast cells isolated from healthy human placentae. Treatment with 8-Br-cAMP likewise increased expression of the syncytiotrophoblast differentiation markers GCM1, hCG-β and Syn-1 (Figures 2c and d). In parallel, 8-Br-cAMP treatment enhanced GCM1 acetylation in BeWo cells and primary villous trophoblast cells (Figures 2e amd f). Importantly, increased syncytiotrophoblast formation was paralleled by reduced p45 NF-E2 protein expression in BeWo cells and in primary villous trophoblast cells (Figures 2c and d). These results firstly demonstrate that human BeWo cells are a suitable *in vitro* model for mechanistic studies evaluating the role of p45 NF-E2 for syncytiotrophoblast differentiation. Secondly, these results demonstrate that excess syncytiotrophoblast formation in human trophoblast cells is associated with a reduced expression of p45 NF-E2, consistent with the proposed role of p45 NF-E2 as a negative regulator of syncytia formation in human trophoblast cells.

### Increased cell death in p45 NF-E2 deficient trophoblast cells

**Figure 3:**
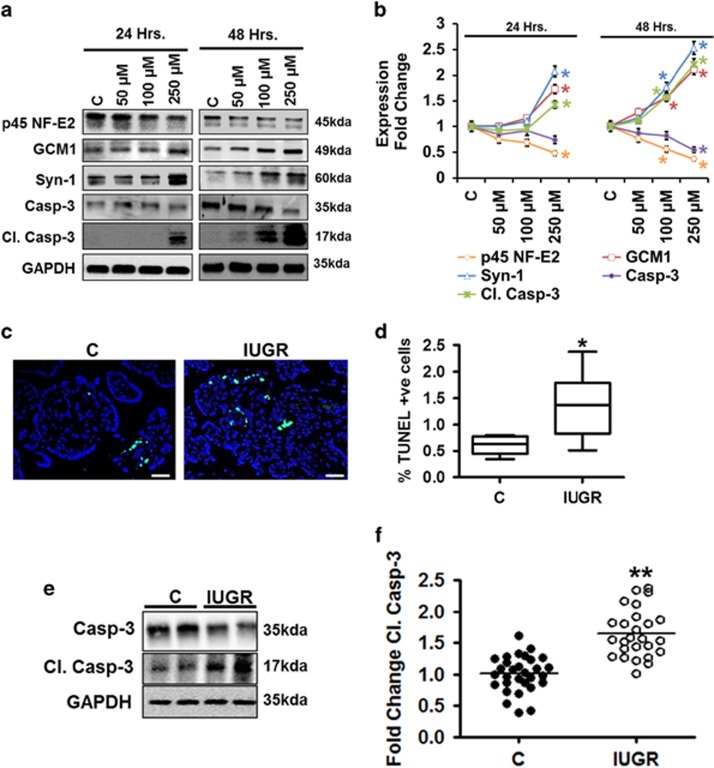
Increased cell death is associated with enhanced syncytiotrophoblast differentiation and reduced p45 NF-E2 expression. (**a** and **b**) Immunoblotting analysis ((**a**) representative immunoblots; (**b**) line graph summarizing results) showing a dose- and time-dependent reduction of p45 NF-E2 in BeWo cells which is paralleled by increased levels of the syncytiotrophoblast differentiation markers GCM1 and Syn-1 and the apoptosis regulator cleaved caspase-3 (Cl. Casp-3). (**c** and **d**) Enhanced frequency of TUNEL-positive cells ((**c**) representative images; (**d**) box-plot summarizing results) in human IUGR placentae indicating enhanced cell death compared with healthy controls. (**e** and **f**) Immunoblotting analysis ((**e**) representative immunoblots; (**f**) bar graph summarizing results) showing enhanced levels of cleaved capsase-3 (Cl. Casp-3) in human IUGR placentae compared with healthy controls. Size bars represent 20 *μ*m (**c**); mean±S.E.M. (**b**,**d** and **f**); results of at least five independent repeat experiments shown (**b**); *P<0.05 (*t*-test in **b**, **d** and **f**)

Activation of apoptosis regulators is closely linked with trophoblast differentiation and turnover.^22, 23, 24^ These processes are physiologically tightly regulated but may be increased in placental diseases. Therefore, we investigated whether p45 NF-E2 deficiency enhances syncytiotrophoblast differentiation and activation of apoptosis regulators. Indeed, 8-Br-cAMP treatment reduced p45 NF-E2 expression, enhanced syncytialization and increased cleaved caspase-3 in a dose- and time-dependent manner *in vitro* (Figures 3a and b). A potential link between reduced p45 NF-E2 expression and increased syncytiotrophoblast differentiation and cell death with IUGR is further corroborated by analyses of human placental samples. Cell death, as indicated by TUNEL, is increased in placentae from IUGR pregnancies as compared with placentae from healthy controls (Figures 3c and d). In parallel, cleaved caspase-3 was increased in tissue lysates obtained from IUGR placentae compared with controls (Figures 3e and f). These *ex vivo* results support the notion that reduced p45 NF-E2 expression is linked with increased cell death and activation of apoptosis regulators, supporting a pathophysiologic relevance of reduced p45 NF-E2 expression in pregnancy complications such as IUGR.

### p45 NF-E2 regulates Gcm1 acetylation in human trophoblast cells

**Figure 4:**
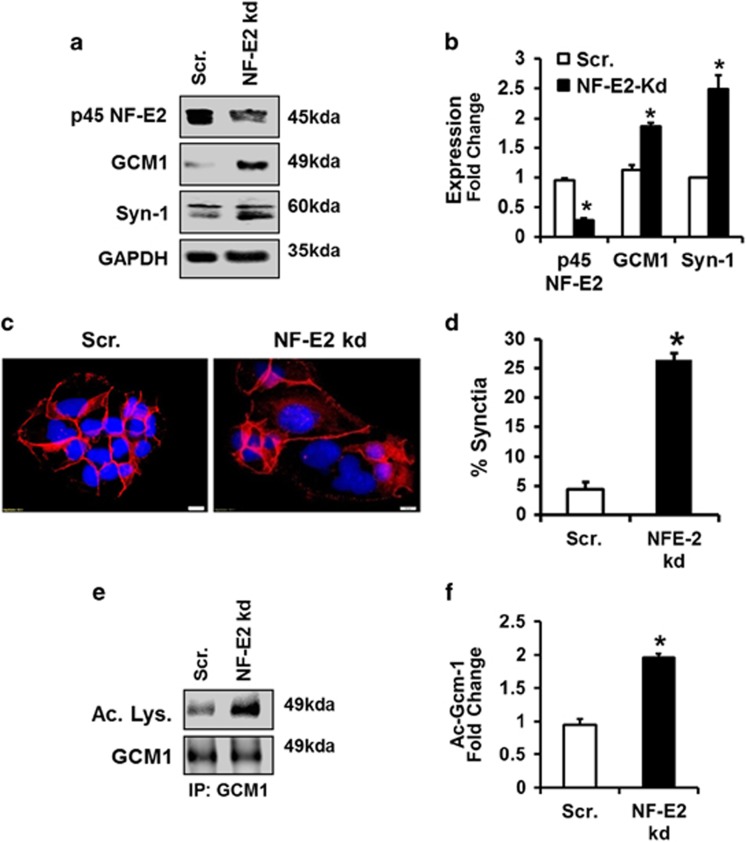
Reduced p45 NF-E2 expression enhances syncytiotrophoblast differentiation in human trophoblast cells. (**a** and **b**) Immunoblotting analysis ((**a**) representative immunoblots; (**b**) bar graph summarizing results) showing a marked reduction of p45 NF-E2 after electroporation of BeWo cells with NF-E2 shRNA. GCM1 and Syn-1 expression is increased after p45 NF-E2 knockdown, reflecting increased syncytiotrophoblast formation. (**c** and **d**) shRNA mediated knockdown of p45 NF-E2 caused enhanced syncytia formation in BeWo cells (immunofluorescence staining; (**c**) representative images; (**d**) bar graph summarizing results; red: E-cadherin; blue: DAPI nuclear staining). (**e** and **f**) p45 NF-E2 knockdown induces acetylation of GCM1. Immunoprecipitation using anti-GCM1 antibody followed by immunoblotting ((**e**) representative immunoblots; (**f**) bar graph summarizing results) showing enhanced GCM1 acetylation after shRNA mediated p45 NF-E2 knockdown in BeWo cells. Size bars represent 20 *μ*m (**a**); mean±S.E.M. (**b**,**d** and **f**); results of at least five independent repeat experiments shown (**b**, **d** and **f**); *P<0.05 (*t*-test in **b**,**d** and **f**)

To evaluate whether syncytium formation in human trophoblast cell line BeWo depends on p45 NF-E2, we next reduced p45 NF-E2 expression in BeWo cells via shRNA knockdown (Figures 4a and b). Knockdown of p45 NF-E2 expression in these cells increased syncytialization, as indicated by increased multinucleate syncytium determined by immunostaining for E-cadherin combined with nuclear DAPI stain (Figures 4c and d). Increased syncytiotrophoblast differentiation was confirmed by an increased expression of Syn-1 and GCM1 expression (Figures 4a and b). Importantly, not only GCM1 expression but also GCM1 acetylation was increased in p45 NF-E2 deficient trophoblast cells (Figures 4e and f).

**Figure 5:**
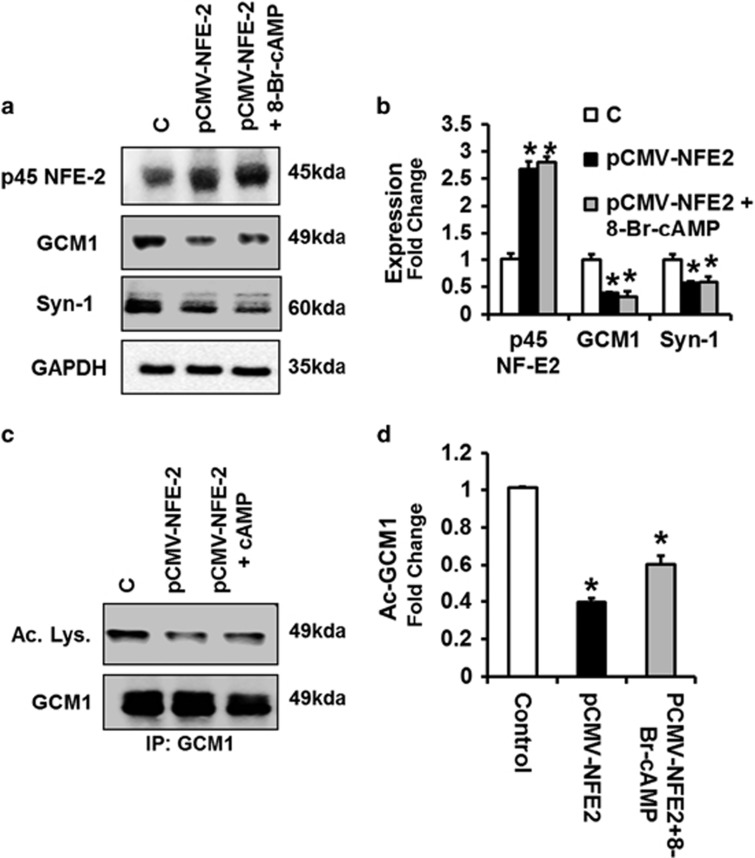
Overexpression of p45 NF-E2 restricts syncytiotrophoblast differentiation in human trophoblast cells. (**a** and **b**) p45 NF-E2 overexpression (pCMV-NF-E2) prevents 8-Br-cAMP induced GCM1 and Syn-1 expression, indicating reduced syncytiotrophoblast formation (immunoblot analysis; (**e**) representative immunoblots; (**f**) bar graph summarizing results). (**c** and **d**) Overexpression of p45 NF-E2 prevents 8-Br-cAMP-induced acetylation of GCM1. Immunoprecipitation using anti-GCM1 antibody followed by immunoblotting ((**g**) representative immunoblots; (**h**) bar graph summarizing results) showing reduced expression of acetylated GCM1 after overexpression of p45 NF-E2. Treatment of BeWo cells overexpressing p45 NF-E2 with 8-Br-cAMP did not increase GCM1 acetylation. Mean±S.E.M. (**b** and **d**); results of at least five independent repeat experiments shown (**b** and **d**); *P<0.05 (ANOVA in **b** and **d**)

To ascertain the causal relevance of p45 NF-E2 in regulating GCM1 acetylation in human trophoblast cells, we next increased p45 NF-E2 expression using a CMV-driven expression construct. Forced expression of p45 NF-E2 was sufficient to repress GCM1 expression (Figures 5a and b) and GCM1 acetylation (Figures 5c and d). Intriguingly, 8-Br-cAMP failed to induce GCM1 expression, GCM1 acetylation and syncytiotrophoblast formation in BeWo cells overexpressing p45 NF-E2 (Figures 5a–d). These observations suggest that p45 NF-E2 is a dominant-negative regulator syncytiotrophoblast differentiation in human trophoblast cells, at least in the employed model.

Taken together, these results show that p45 NF-E2 regulates GCM1 expression, GCM1 acetylation and syncytiotrophoblast differentiation in a human trophoblast cell line. However, the mechanism through which the transcription factor p45 NF-E2 regulates GCM1 acetylation and syncytiotrophoblast differentiation remains unknown.

### p45 NF-E2 regulates syncytiotrophoblast differentiation by CBP-mediated acetylation of GCM1

The effect of GCM1 in trophoblast differentiation is regulated through various post-translational modifications. Thus, its stability is regulated at least in part by CBP-mediated acetylation. We therefore investigated whether p45 NF-E2 regulates GCM1 acetylation and expression via CBP in BeWo cells and in human placentae.

**Figure 6:**
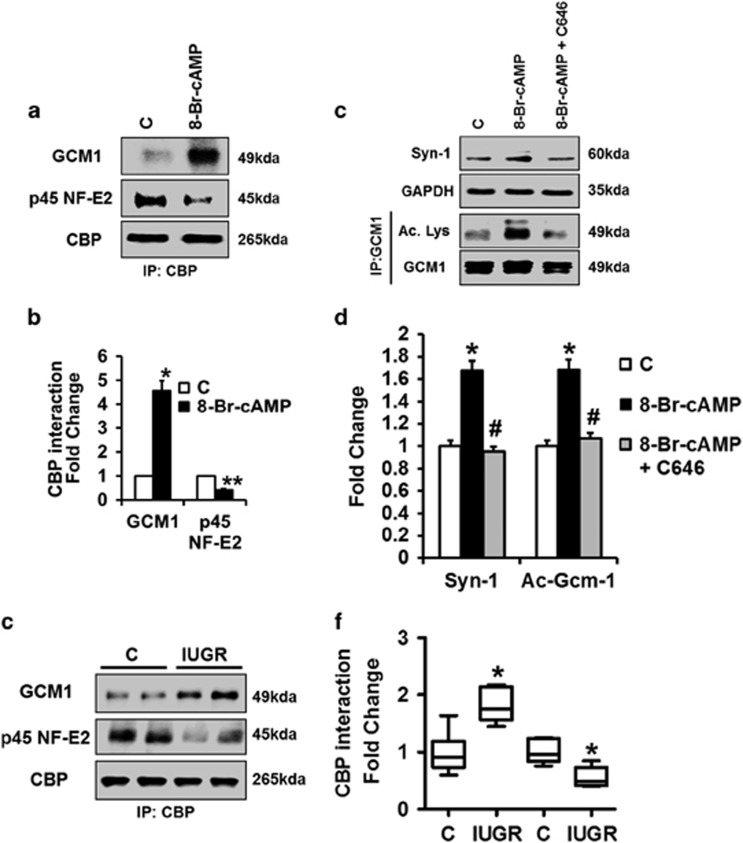
p45 NF-E2 regulates syncytiotrophoblast differentiation by CBP-mediated GCM1 acetylation in human trophoblast cells. (**a** and **b**) Showing CBP and p45 NF-E2 interaction is reduced but CBP and GCM1 interaction is increased after treatment of BeWo cells with 8-Br-cAMP; immunoprecipitation using the anti-CBP antibody followed by immunoblotting ((**a**) representative immunoblots; (**b**) bar graph summarizing results). (**c** and **d**) C646 (inhibitor of CBP acetyltransferase activity) reduces expression of Syn-1 (immunoblotting; (**c**) representative immunoblots; (**d**) bar graph summarizing results) and prevents GCM1 acetylation (immunoprecipitation using the anti-GCM1 antibody followed by immunoblotting; (**c**) representative immunoblots; (**d**) bar graph summarizing results). (**e** and **f**) Reduced p45 NF-E2-CBP interaction but enhanced CBP–GCM1 interaction in placenta tissue lysates obtained from women with pregnancies complicated with IUGR compared with controls; immunoprecipitation using anti-CBP antibody followed by immunoblotting ((**e**) representative immunoblots; (**f**) box-plot summarizing results). Mean±S.E.M. (**b**, **d** and **f**); results of at least five independent repeat experiments shown (**b**, **d** and **f**); *P<0.05 (*t*-test in **b** and **f**; ANOVA in **d**)

Using the above *in vitro* trophoblast differentiation model we first analyzed the impact of syncytiotrophoblast formation on the interaction of CBP with GCM1 and p45 NF-E2. In untreated control cells a strong interaction of CBP with p45 NF-E2 was observed, while that of CBP with GCM1 was negligible (Figures 6a and b). Following induction of syncytiotrophoblast differentiation using 8-Br-cAMP the interaction of CBP with p45 NF-E2 markedly decreased, while that with GCM1 markedly increased (Figures 6a and b). This interaction pattern of CBP with p45 NF-E2 and GCM1 indicates that p45 NF-E2 may reduce GCM1 acetylation by inhibiting its interaction with CBP, which possesses acetyltransferase activity. Indeed, inhibiting the acetyltransferase activity of CBP using C646 normalized GCM1 acetylation and Syn-1 expression in the 8-Br-cAMP model (Figures 6c and d). We next analyzed the interaction of CBP with GCM1 and p45 NF-E2 in human placentae complicated with IUGR and healthy controls. In IUGR placentae the interaction between CBP and p45 NF-E2 was reduced, while that between CBP and GCM1 was increased (Figures 6e and f). These observations made with human tissue samples corroborate the relevance of the above *in vitro* results. Based on these data we propose that p45 NF-E2 inhibits GCM1 acetylation by preventing the interaction of GCM1 with CBP.

### p45 NF-E2 regulates syncytiotrophoblast differentiation by SENP-mediated desumoylation of GCM1

**Figure 7:**
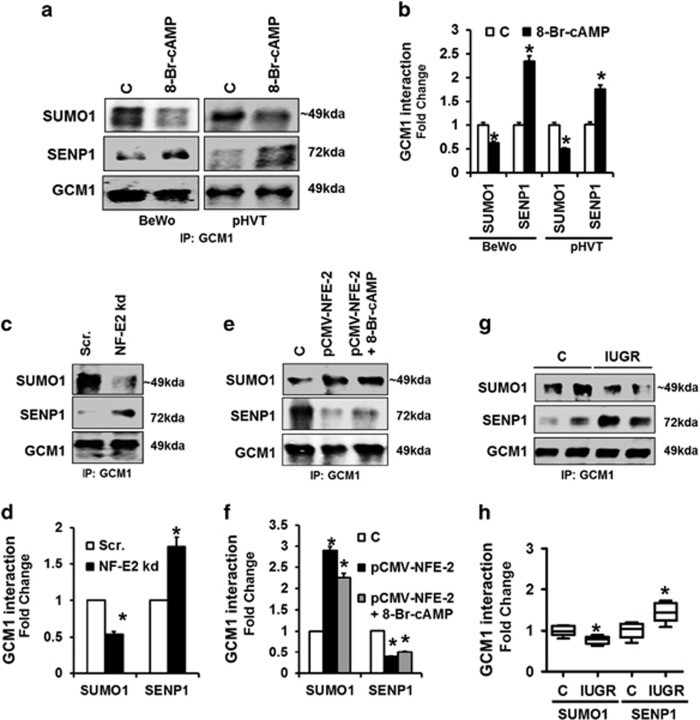
p45 NF-E2 regulates syncytiotrophoblast differentiation by SENP1-mediated GCM1 desumoylation in human trophoblast cells. (**a**–**d**) Decreased GCM1 sumoylation and enhanced GCM1–SENP1 interaction in 8-Br-cAMP-treated BeWo cells and primary human villous trophoblast cells (pHVT; **a** and **b**) and p45 NF-E2-deficient (**c** and **d**) BeWo cells; immunoprecipitation using the anti-GCM1 antibody followed by immunoblotting ((**a** and **c**) representative immunoblots; (**b** and**d)** bar graph summarizing results). (**e** and **f**) Overexpression of p45 NF-E2 induces GCM1 sumoylation while reducing the GCM1–SENP1 interaction both in the absence or presence of 8-Br-cAMP; immunoprecipitation using the anti-GCM1 antibody followed by immunoblotting ((**e**) representative immunoblots; (**f**) bar graph summarizing results). (**g** and **h**) Reduced GCM1 sumoylation and enhanced SENP1-GCM1 interaction in placenta tissue lysates obtained from women with pregnancies complicated with IUGR compared with controls, immunoprecipitation using anti-GCM1 antibody followed by immunoblotting ((**d**) representative immunoblots; (**e**) box-plot summarizing results). Mean±S.E.M. (**b**, **d**,**f and h**); results of at least five independent repeat experiments shown (**b**, **d** and **f**); *P<0.05 (*t*-test in **b**,**d** and **h**; ANOVA in **f**)

While GCM1 stability is regulated by its acetylation, DNA-binding activity is regulated by SENP1-mediated desumoylation. As shown above (Figures 4a and b) we observed increased Syn-1 expression and syncytiotrophoblast formation in BeWo cells with reduced p45 NF-E2 expression, indicating increased DNA-binding activity of GCM1 and hence desumoylation. First, we ascertained that GCM1 interacts with SENP1, resulting in desumoylation of GCM1 in the 8-Br-cAMP syncytiotrophoblast differentiation model. Indeed, following exposure of BeWo cells to 8-Br-cAMP we observed an increased interaction of GCM1 with SENP1, which was associated with a reduced sumoylation of GCM1 (Figures 7a and b). The increased interaction of GCM1 with SENP and reduced sumoylation of GCM1 upon exposure to 8-Br-cAMP were confirmed in primary villous trophoblast cells isolated from healthy human placentae (Figures 7a and b).

To determine whether p45 NF-E2 regulates the interaction of GCM1 with SENP1 and GCM1 desumoylation, we next evaluated p45 NF-E2 knocked-down BeWo cells. A reduced expression of p45 NF-E2 enhanced the interaction of GCM1 and SENP1, which was associated with GCM1 desumoylation (Figures 7c and d). Conversely, in BeWo cells with increased p45 NF-E2 expression (CMV-driven p45 NF-E2 overexpression) GCM1–SENP1 interaction was reduced, while sumoylation of GCM1 was increased (Figures 7e and f). This effect was sustained even after treatment with 8-Br-cAMP, again indicating that p45 NF-E2 has a dominant effect in this model (Figures 7e and f).

**Figure 8:**
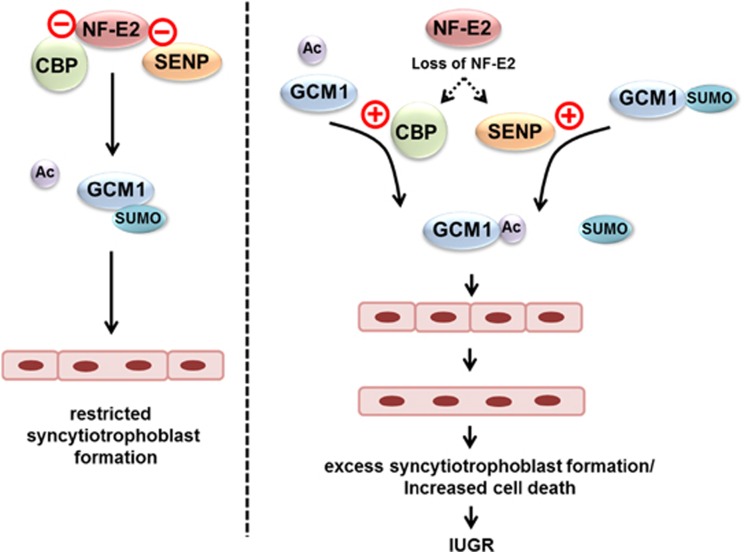
Proposed model**.** In unperturbed trophoblast cells p45 NF-E2 prevents Gcm1 acetylation and desumoylation by CBP and SENP, respectively, thus restricting excess Gcm1-mediated syncytiotrophoblast formation. Loss of p45 NF-E2, as observed in human IUGR, results in enhanced CBP-mediated Gcm1 acetylation and SENP-mediated Gcm1 desumoylation, allowing excess syncytiotrophoblast differentiation which promotes placental insufficiency. These processes are associated with enhanced cell death in IUGR placentae

These *in vitro* studies suggest that p45 NF-E2 does not only regulate GCM1 acetylation but also GCM1 desumoylation, which is an important determinant of GCM1 DNA-binding activity.^25^ To evaluate the potential translational relevance of these findings, we next analyzed the human placentae obtained from pregnancies complicated by IUGR *versus* healthy controls. Indeed, reduced p45 NF-E2 expression was not only associated with increased GCM1 acetylation as shown above (Figures 1f and g) but also with desumoylation of GCM1 in placentae obtained from pregnancies complicated by IUGR (Figures 7g and h). Desumoylation of GCM1 was associated with an increased interaction of SENP1 with GCM1 in human placentae obtained from pregnancies complicated by IUGR (Figures 7g and h). These results demonstrate a negative interaction between acetylation and sumoylation of GCM1 in IUGR placentae, which – based on the *in vitro* studies and the *ex vivo* analysis of human placentae – appears to be regulated by p45 NF-E2 expression (Figure 8). These data suggest that in human placenta disease GCM1 is primarily regulated by post-translational modifications and that p45 NF-E2 negatively regulates GCM1 activity and syncytiotrophoblast formation through a coordinated regulation of GCM1 acetylation and desumoylation.

## Discussion

IUGR remains a medical challenge, due to the lack of suitable biomarkers and therapeutics. This reflects a paucity of mechanistic insights. Within the current study we identify a potential mechanism impairing trophoblast differentiation and causing IUGR in humans. Extending on studies in genetically modified mice^18^ we first establish that expression of the transcription factor p45 NF-E2 is reduced in human pregnancy complicated by IUGR. Using a combination of *ex vivo* analysis employing human placental tissues and *in vitro* models using a human trophoblast cell line, we demonstrate that p45 NF-E2 regulates syncytiotrophoblast differentiation through post-translational modifications of GCM1. Intriguingly, p45 NF-E2 regulates both acetylation and sumoylation of GCM1, suggesting that p45 NF-E2 is a master regulator of GCM1 in human trophoblast differentiation and placentation.

The role of GCM1 for trophoblast function and differentiation is well studied *in vitro* and *in vivo*^9, 19, 20, 26^ and is supported by altered GCM1 expression in human placenta from women with pregnancy complications such as IUGR or preeclamspia.^8, 9^ Furthermore, *in vitro* studies support a mechanistic relevance of post-translational GCM1 modifications for syncytiotrophoblast formation.^26, 27^ However, physiological regulators of post-translational GCM1 modifications and their relevance in human placental diseases remain largely unknown. We demonstrate that expression of p45 NF-E2, which regulates both GCM1 acetylation and sumoylation, is closely related to the post-translational modification of GCM1 in human placentae without or with IUGR. Furthermore, *in vitro* analysis of human trophoblast like cells illustrates the functional relevance of p45 NF-E2 for GCM1 acetylation and sumoylation and for the expression of GCM1-dependent genes. Thus, the current study does not only corroborate the *in vivo* relevance of previous work by others and us,^18, 19, 26^ but in addition identifies a (patho-) physiological relevance of p45 NF-E2 and post-translational modifications of GCM1 in human trophoblast cells and placental disease.

*In vitro* analyses suggest that endometrial cells (RL-95 cells) do not express p45 NF-E2, while immunohistochemical analyses indicates weak expression of p45 NF-E2 in the endometrium (data not shown and human protein atlas; www.proteinatlas.org). Based on the immunohistochemical analyses a function of p45 NF-E2 not only in trophoblast cells but also in maternal cells at the feto-maternal interface cannot be excluded. However, careful analyses in mice established that the absence of p45 NF-E2 in trophoblast cells, but not in maternal cells, conveys the IUGR.^18^ Future studies are needed to decipher whether maternal p45 NF-E2 expression at the feto-maternal interface is of functional relevance.

The current work establishes that p45 NF-E2 mediates GCM1 acetylation via CBP, whereas a recent *in vitro* study demonstrated that HDAC5 conveys deacetylation of GCM1.^28^ In addition to p45 NF-E2 other negative GCM1 regulators are GATA3 and caspase-14^26, 29^ and several post-translational modifications controlling GCM1 stability and/or activity have been identified.^13, 19, 20^ Collectively, these findings underscore the importance of a tight positive and negative regulation of GCM1 activity through post-translational modifications. Notably, we demonstrate that altered p45 NF-E2 expression and GCM1 activity is associated with impaired placental function in mouse and human placenta^18^ (this study), underscoring the *in vivo* relevance and the potential implications for human placental disease of the proposed p45 NF-E2-dependent regulation of trophoblast differentiation.

While our work focused on the role of p45 NF-E2 in trophoblast differentiation and placental function, others have investigated the function of p45 NF-E2 during hematopoiesis, in particular megakaryopoiesis.^15^ Congruently with our observations in trophoblast cells p45 NF-E2 regulates H3 acetylation during hematopoiesis,^30^ suggesting that p45 NF-E2-dependent regulation of protein acetylation is of broader relevance. Of note, during both syncytiotrophoblast differentiation and megakaryopoiesis multinuclear cells are generated. While the mechanisms differ largely,^31, 32^ the question arises whether p45 NF-E2 regulates a common aspect in the process of multinuclear cell development.

Trophoblast differentiation is closely linked with temporally and spatially regulated activation of apoptosis regulators and cell death induction.^22, 23, 24^ In accordance with a function of p45 NF-E2 in regulating trophoblast differentiation, we observed increased caspase-3 activation and cell death in association with reduced p45 NF-E2 expression *in vitro* and *in vivo*. This finding is congruent with the increased frequency of syncytial knots in IUGR.^24, 33, 34^ Further experiments are required to ascertain the mechanistic link between reduced p45 NF-E2 expression, increased activation of pro-apoptotic regulators and altered trophoblast differentiation.

The functional consequence of the p45 NF-E2-dependent GCM1 regulation for placental dysfunction in human patients with IUGR remains to be established. IUGR is generally thought to be caused by impaired placental perfusion.^35, 36^ Unfortunately, data on placental flow prior to delivery were not available to us in the present study. Prospective studies are required to evaluate the potential association between placental p45 NF-E2 and GCM1 expression, GCM1 post-translational modifications and placental perfusion. Furthermore, the potential association of altered p45 NF-E2 expression and GCM1 post-translational modifications with biomarkers and regulators of placental vascularization (e.g. vascular endothelial growth factor, placental growth factor) should be investigated in future studies. Intriguingly, GCM1 regulates trophoblast proliferation and differentiation and we previously observed altered differentiation of various trophoblast subtypes in mice with p45 NF-E2 deficiency, corroborating the functional link between p45 NF-E2, GCM1 and trophoblast differentiation.^10, 18^

Additionally, reduced GCM1 expression has been linked with increased tissue inhibitor of metalloproteinase-4 (TIMP4) expression in preeclampsia,^37^ indicating a mechanistic link between GCM1, placental tissue-remodeling and placental vascularization. Whether reduced p45 NF-E2 expression is linked with altered matrix-metalloproteinase activity remains to be evaluated in future studies.

Unlike in the mouse model and in the murine and human *in vitro* models, we did not observe an induction of GCM1 expression (protein level) in human placenta tissue biopsies. This may have several potential reasons. The etiology of IUGR is multifarious and the proposed mechanism may be of pathophysiological relevance in a subset of IUGRs only.^38^ Accordingly, we observed elevated GCM1 expression in a subgroup of placenta tissues (see Figure 1e). GCM1 expression levels may hence enable to distinguish different subgroups of IUGR. Alternatively, the differences observed in mice and *in vitro* compared with the human tissues imply that in humans GCM1 is primarily regulated at the post-translational level. Accordingly, analyses of GCM1 post-translational modifications may be more informative than analyses of its expression level when studying human placental dysfunction. Future studies carefully characterizing the placenta tissues, the disease course and other biomarkers are required to clarify whether GCM1 enables stratification of IUGR subgroups and whether post-translational modifications of GCM1 are more suitable than total expression levels for diagnostic purposes.

IUGR may severely impact the health of the newborn^39^ and neurodevelopmental impairment manifests itself early and typically before clinical decision making.^40^ The current results do not only propose a new mechanism (Figure 8) causing placental dysfunction and IUGR but may lay ground for the development of new biomarkers allowing early detection of IUGR.

## Materials and methods

### Materials

The following antibodies were used in the current study: goat *α*GCM1, rabbit *α*NLRP3, mouse *α*CBP (Santa Cruz, Heidelberg, Germany), rabbit *α*-acetylated lysine, rabbit *α*SUMO1, rabbit *α*SENP1, rabbit *α*-E-cadherin (New England Biolabs, Frankfurt am Main, Germany), rabbit *α*GAPDH (Sigma Aldrich, St. Louis, USA). The following HRP conjugated secondary antibodies were used for immunoblotting: goat *α*-rabbit IgG-HRP, horse *α*-mouse IgG-HRP (New England Biolabs) and donkey *α*-goat IgG-HRP (Santa Cruz). The following secondary antibodies for immunofluorescence were used: TRITC conjugated swine-*α*-rabbit (Dako, Glostrup, Denmark).

Other reagents used in the current study were: 8-Br-cAMP, pencillin–Streptomycin (Sigma Aldrich), protein A/G agarose beads, C646 (Santa Cruz), electroporation cuvettes (Peqlab, Germany), *Eco*RI, *Xho*I, *Sac*I restriction enzymes (New England Biolabs), Go Taq Polymerase, T4 DNA ligase (Promega, Mannheim, Germany), bicinchonic acid (BCA) reagent, pOTB7-NF-E2 cDNA plasmid and pLKO1-NF-E2 knockdown plasmids (Thermofischer Scientific, Darmstadt, Germany), Ham's F-12K, Trypsin-EDTA, fetal bovine serum and HEPES (Gibco, Darmstadt, Germany; Thermofischer Scientific); protease inhibitor cocktail (Roche Diagnostics GmbH, Mannheim, Germany); Vectashield mounting medium with DAPI, (Vector Laboratories, Burlingame, CA, USA); polyvinylidene fluoride (PVDF) membrane and immobilion enhanced chemiluminescence reagent (Millipore GmbH, Darmstadt, Germany).

### Human placenta samples

Human placenta samples from pregnancies complicated with normotensive IUGR and normotensive control pregnancies were provided by the Frauenklinik, Universitätsklinikum Erlangen (Erlangen, Germany), Universitätsfrauenklinik, Magdeburg and the Klinkum Olvenstedt, Magdeburg (Magdeburg, Germany) in accordance with the guidelines and with the approval of the local ethics committee and after obtaining informed consent of patients. IUGR was defined as fetal growth below the 5% percentile. Clinical characteristics of patients are shown in Supplementary Table S1.

### *In vitro* interventions, knockdown and overexpression

BeWo cells, human trophoblast like cells with fusogenic potential, were obtained from ATCC (Middlesex, UK) and were cultured at 37 °C in a humidified incubator with 5% CO_2_ in Ham's F-12K nutrient mixture with 10% fetal bovine serum and 1% pencillin–streptomycin. Primary villous trophoblast cells obtained from healthy human placenta were obtained from ScienCell Research Laboratories (Carlsbad, CA, USA) and cultured at 37 °C in a humidified incubator with 5% CO_2_ in trophoblast medium with supplements as recommended by the provider.^41^ To study the role of the transcription factor p45 NF-E2 in syncytiotrophoblast formation, BeWo cells or primary trophoblast cells were treated with 250 *μ*M 8-Br-cAMP to induce differentiation into syncytiotrophoblast. For cell death experiments, Bewo cells were treated with increasing concentrations of 8-Br-cAMP for 24 and 48 h. In some experiments, BeWo cells were treated with 8-Br-cAMP along with 5 *μ*M C646 (CBP acetyltransferase inhibitor). Knockdown and overexpression of p45 NF-E2 was done using electroporation of plasmids as described below.

Human p45 NF-E2 (pLKO1-NF-E2) knockdown constructs containing shRNA for p45 NF-E2 was purchased from Thermoscientific. The p45 NF-E2-expressing plasmid pCMV-NF-E2-cMyc was generated by sub-cloning pOTB7-NF-E2 under the regulation of CMV promoter. In detail, a 260bp PCR fragment was amplified from pOTB7-NF-E2 using a forward primer carrying an *Eco*RI overhang and located at the p45 NF-E2 start codon (forward, mutated to GGG, CGAATTCACGGGTCCCCGTGT) and a reverse primer containing an endogenous *Sac*I site (GATGCTGGGAGCTCATAAGG, 240 bp downstream of transcription start site). Forty nanograms pOTB7-NF-E2 cDNA was used as a template and the resulting 260 bp amplimer (initial denaturation: 95 °C–10 min, 35 cycles of denaturation: 95 °C–30 s, annealing: 60 °C–30 s, extension: 72 °C–25 s, final extention: 72 °C–5 min) was cloned into a TOPO vector and then cloned back into pOTB7-NFE-2 using *Eco*RI/*Sac*I digest. The gene sequence containing the mutated start site was then digested with *Eco*RI/*Xho*I and cloned into the pCMV-cMyc vector, generating the p45 NF-E2 overexpressing construct. Plasmids were electroporated into BeWo cells using a single high-voltage pulse of 250 V and 960 *μ*F (BTX Electroporator, Holliston, MA, USA).

### Histology

Tissues were fixed in 4% buffered paraformaldehyde for 2 days, embedded in paraffin and processed for sectioning. Placental morphology was analyzed on H&E stained sections. Human placentae sections were studied for syncytial knot formation. In each section at least 10 randomly selected microscopic fields from three non-consecutive placental sections (magnification × 40) were acquired. Syncytial knots were identified as aggregates of at least 10 syncytial nuclei protruding at the surface of terminal villi but not in contact with adjacent villi.^42^ Trophoblast villous area in mm^2^ was calculated for these images using thresholding as explained earlier^43^ and number of syncytial knots per mm^2^ of villus was determined.

### Immunostaining

Immunofluorescence was performed on human placenta sections for p45 NF-E2 and on BeWo cells for E-Cadherin (to assess *in vitro* syncytia formation). Briefly, sections were de-paraffinized and hydrated followed by post-fixation in ice cold acetone for 1 min and washing in PBS for 10 min. Antigen retrieval was performed using antigen unmasking solution (Vector) and unspecific peroxidase activity was blocked by incubating the section in 3% H_2_O_2_. For *in vitro* immunofluorescence, cells were fixed with ice cold methanol for 10 min followed by PBS wash for 2 × 5 min. This was followed by blocking (blocking solution: 1.5% serum, 3% BSA prepared in PBS containing 0.05% Tween-20) for 1 h. The sections or cells were then incubated overnight at 4 °C with primary antibodies against p45 NF-E2 or E-Cadherin, respectively. Following washing in PBS for 10 min, sections/cells were incubated for 120 min with corresponding fluorophore-labeled secondary antibody. Sections/cells incubated without primary antibodies were used as negative controls. Sections/cells were then rinsed twice for 10 min each in PBS and mounted in Vectashield containing DAPI and visualized using a fluorescence microscope. Image exposure and acquisition settings were set using negative controls (without primary antibodies) and identical settings were used for all sections/cell treatments. Multinucleate cells (containing more than three nuclei) were regarded as one syncytia. *In vitro* syncytia formation was calculated as a percentage ratio between the number of nuclei within the syncytia and number of nuclei outside the syncytia.

### Immunoblotting

Cell lysates were prepared using radio immunoprecipitation assay (RIPA) buffer containing 50 mM Tris (pH 7.4), 1% NP-40, 0.25% sodium-deoxycholate, 150 mM NaCl, 1 mM EDTA, 1 mM Na_3_VO_4_, 1 mM NaF supplemented with protease inhibitor cocktail. For tissue lysates, RIPA with 0.5% sodium-deoxycholate was used. Lysates were centrifuged (13 000 × *g* for 10 min at 4 °C) and insoluble debris was discarded. Protein concentration in supernatants was quantified using BCA reagent. Equal amounts of protein were electrophoretically separated on 7.5, 10 or 12.5% SDS polyacrylamide gel, transferred onto PVDF membranes and probed with desired primary antibodies overnight at 4 °C. Membranes were then washed with PBST and incubated with anti-mouse IgG (1:5000) or anti-rabbit IgG (1:2000) horseradish peroxidase-conjugated antibodies as indicated. Blots were developed with the enhanced chemiluminescence system. To compare and quantify levels of proteins, the density of each band was measured using Image J software (National Institutes of Health, Bethesda, MD, USA). Equal loading for total cell or tissue lysates was determined by glyceraldehyde 3-phosphate dehydrogenase western blot.

### Immunoprecipitation

Immunoprecipitation for GCM1 was carried out in a total volume of 500 *μ*l containing 200 *μ*g of cell lysate diluted in RIPA buffer. Lysates were incubated with 1 *μ*g of anti-GCM1 antibody for 4 h at 4 °C while rotation. Twenty microlitres of protein A/G agarose beads were then added and incubated further for overnight at 4 °C while rotation. Immunoprecipitates conjugated to beads were collected by centrifugation at 12 000 × *g* for 30 s. They were then washed with 1 ml RIPA buffer each for three times at 3000 × *g* for 5 min each. Immunoprecipitates were eluted from the beads by addition of 1 × Laemelli's buffer and boiling the samples at 95 °C for 10 min. Beads were separated by centrifugation and immunoprecipitates were analyzed by immunoblotting. Host-derived IgG was used as a control for immunoprecipitation (Supplementary Figure S1).

### Statistical analysis

The data are summarized as the mean±S.E.M. Statistical analyses were performed with Student's *t*-test, *χ*^2^ test, Spearman's correlation, Mann–whitney *U*-test or ANOVA as appropriate. *Post hoc* comparisons of ANOVA were corrected with the method of Tukey. The Kolmogorov–Smirnov test or D'Agostino-Pearson normality-test was used to determine whether the data are consistent with a Gaussian distribution. Prism 5 (www.graphpad.com) software was used for statistical analyses. Statistical significance was accepted at *P*-values of <0.05.
