# Supplementary material for: p45 NF-E2 regulates syncytiotrophoblast differentiation by post-translational GCM1 modifications in human intrauterine growth restriction
Source: Cell Death Dis. 2017 Apr 6;8(4):e2730–. doi: 10.1038/cddis.2017.127 (PMC5477575; doi:10.1038/cddis.2017.127)
Supplement: Supplementary Information [file cddis2017127x1.pdf]

	Controls		IUGR		
	Mean	± SD	Mean	SD	p
N	30		26		NA
Age (years)	32.67	± 4.54	29.0	± 5.87	0.32
BMI (kg/m2)	27.67	± 4.13	24.14	± 3.73	0.81
Systolic blood pressure (mmHg)	123.00	± 8.52	124.61	± 7.71	0.51
Diastolic blood pressure (mmHg)	75.67	± 2.20	78.84	± 7.50	0.14
Gestational diabetes	0.00	0.00	0.00	0.00	NA
Proteinuria	0.00	0.00	0.00	0.00	NA
Gestational age at birth	39.08	± 1.09	38.04	± 1.86	0.06
Relative birth weight	3517.08	± 357.13	2153.8	± 419.58	<0.001

Table-1: Clinical characteristics of human placenta samples collected at term from healthy control pregnancies (n=30) and pregnancies complicated with normotensive intrauterine-growth restriction (IUGR, n=26, growth <5th percentile) pregnancies. NA: not applicable
