# Supplementary material for: p45 NF-E2 regulates syncytiotrophoblast differentiation by post-translational GCM1 modifications in human intrauterine growth restriction
Source: Cell Death Dis. 2017 Apr 6;8(4):e2730–. doi: 10.1038/cddis.2017.127 (PMC5477575; doi:10.1038/cddis.2017.127)
Supplement: Supplementary Information [file cddis2017127x2.pdf]

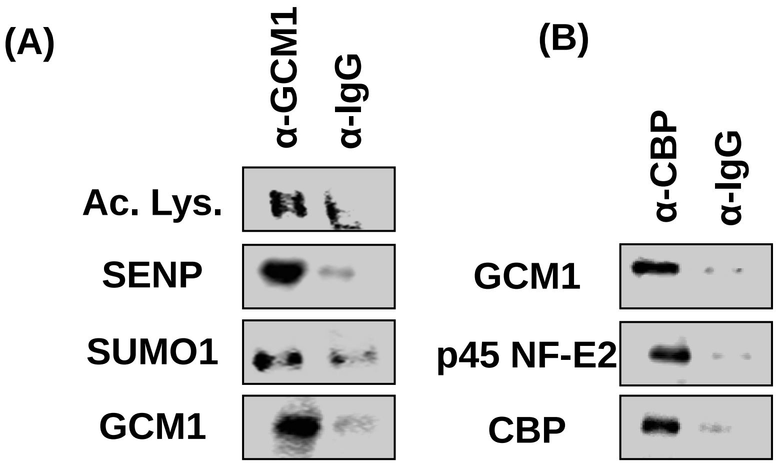

Supplementary Fig. 1: Representative immunoblots after immunoprecipitation with Gcm1 (A) or CBP (B) along with respective IgG as controls showing specific pull down for GCM1 (A) and CBP (B) respectively.
