# Supplementary material for: p45 NF-E2 regulates syncytiotrophoblast differentiation by post-translational GCM1 modifications in human intrauterine growth restriction
Source: Cell Death Dis. 2017 Apr 6;8(4):e2730–. doi: 10.1038/cddis.2017.127 (PMC5477575; doi:10.1038/cddis.2017.127)
Supplement: Supplementary Information [file cddis2017127x3.pdf]

Detailed Author Contributions

Figure-1: M.M.A performed and analyzed H&E staining (1A,B), K.S. did immunostaining (1C), S.K. performed expression analysis (1D-E), J.M. performed and analyzed immunoprecipitation (1F,G), H.H, J.H, F.B.F, M.L., A.C.Z., M.R. provided placenta samples used in 1A-G, B.I. interpreted the data in 1A-G.

Figure-2: I.G. did cell culture and immunostaining (2A,B), S.K. performed expression analysis and immunoprecipitation (2C-F), A.A and B.B helped in experimental planning and data interpretation (2A-F), B.I. interpreted the data in 2A-F.

Figure-3: S.K. did cell culture and immunoblotting (3A,B), P.M. did TUNEL staining and analysis (3C,D), F.L. did immunoblotting for human samples (3E,F). H.H, J.H, F.B.F, M.L., A.C.Z., M.R. provided placenta samples used in 3C-F.

Figure-4: S.K. did knockdown, expression analysis and immunoprecipitation (4A-F), K.S. did cell culture and immunostaining (4C,D), A.A and B.B helped in experimental planning and data interpretation (4A-F), B.I. interpreted the data in 3A-F.

Figure-5: S.K. did cloning, transfection, expression analysis and immunoprecipitation experiments (5A-D), A.A and B.B helped in experimental planning and data interpretation (5A-D), B.I. interpreted the data in 5A-D.

Figure-6: S.K. performed immunoprecipitation experiments and expression studies (6A-F), I.G. did cell culture and treatments (6C-D), H.H, F.B.F, J.H, M.L., A.C.Z., M.R. provided placenta samples used in 5E,F, B.I. interpreted the data in 6A-G.

Figure-7: S.K. performed immunoprecipitation experiments and expression studies (7A-H), J.M. assisted in immunoprecipitation analysis (7H), H.H, F.B.F, J.H, M.L., A.C.Z., M.R. provided placenta samples used in 6G,H, B.I. interpreted the data in 7A-H.
